# Coccidioidomycosis among Workers at an Archeological Site, Northeastern Utah

**DOI:** 10.3201/eid1004.030446

**Published:** 2004-04

**Authors:** Lyle R. Petersen, Stacie L. Marshall, Christine Barton, Rana A. Hajjeh, Mark D. Lindsley, David W. Warnock, Anil A. Panackal, Joseph B. Shaffer, Maryam B. Haddad, Frederick S. Fisher, David T. Dennis, Juliette Morgan

**Affiliations:** * Centers for Disease Control and Prevention, Ft. Collins, Colorado, USA; † Utah Department of Health, Salt Lake City, Utah, USA; ‡ Centers for Disease Control and Prevention, Atlanta, Georgia, USA; § TriCounty Health Department, Vernal, Utah, USA; ¶ University of Arizona, Tucson, Arizona, USA

**Keywords:** Coccidioidomycosis, pulmonary, epidemiology, outbreak, *Coccidioides immitis*

## Abstract

In 2001, an outbreak of acute respiratory disease occurred among persons working at a Native American archeological site at Dinosaur National Monument in northeastern Utah. Epidemiologic and environmental investigations were undertaken to determine the cause of the outbreak. A clinical case was defined by the presence of at least two of the following symptoms: self-reported fever, shortness of breath, or cough. Ten workers met the clinical case definition; 9 had serologic confirmation of coccidioidomycosis, and 8 were hospitalized. All 10 were present during sifting of dirt through screens on June 19; symptoms began 9–12 days later (median 10). Coccidioidomycosis also developed in a worker at the site in September 2001. A serosurvey among 40 other Dinosaur National Monument workers did not find serologic evidence of recent infection. This outbreak documents a new endemic focus of coccidioidomycosis, extending northward its known geographic distribution in Utah by approximately 200 miles.

Coccidioidomycosis results from inhaling spores (arthroconidia) of *Coccidioides immitis,* a soil-dwelling fungus endemic to the southwestern United States and parts of Mexico, Central America, and South America. The organism is recovered from soil in areas with yearly annual rainfall averaging 5–20 inches, hot summers, infrequent winter freezes, and alkaline soil (*1*). Although up to 100,000 new infections may occur annually (*2*), reported point source outbreaks are infrequent and have followed diverse soil-disrupting activities or events, such as archeological or anthropological digs (*3*–*5*), military maneuvers (*6*), play involving throwing dirt (*7*), construction work (*8*), earthquakes (*9*), dust storms (*10*,*11*), model airplane flying competitions (*12*), and armadillo hunting (*13*).

We report a point-source outbreak of coccidioidomycosis among workers who participated in soil-disrupting activities at an archeological site in Dinosaur National Monument in northeastern Utah during June and July 2001. This site was approximately 200 miles north of previously known *C. immitis–*endemic areas for in Utah (Figure 1). In addition, we report results of a serologic survey of Dinosaur National Monument employees performed to assess recent exposure to *C. immitis*.

**Figure 1:**
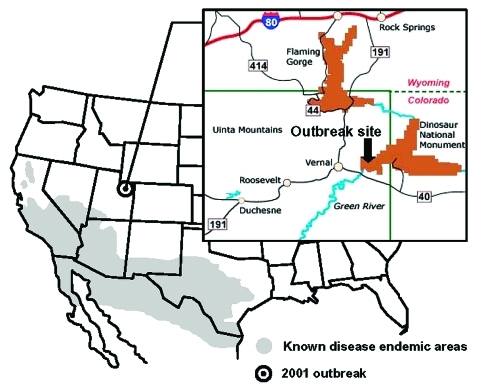
Known geographic distribution of *Coccidioides immitis* in the United States and location of the 2001 coccidioidomycosis outbreak in Utah. Source: U.S. Geological Survey, Operational Guidelines for Geological Fieldwork in Areas Endemic for Coccidioidomycosis (Valley fever), 2000.

## Outbreak Setting

Dinosaur National Monument covers 320 square miles in the Uinta Basin in northeastern Utah and northwestern Colorado (Figure 1). A total of 397,800 visitors were recorded in 2000. During summer 2001, 49 permanent and 49 seasonal employees, as well as approximately 120 volunteers, worked at the monument. A weather station, located approximately 0.5 miles from the outbreak site, has recorded an average annual precipitation of 8.7 inches since 1958.

The outbreak site, at an elevation of 4,825 feet in an arid, treeless region with small hills and rock outcroppings, is under a rock overhang. The overhang faces directly south and receives reflected heat from the surrounding frontier sandstone. Heat trapped within the shelter raises the temperature several degrees above the outside temperature, hence its name, Swelter Shelter (*14*). Swelter Shelter’s soil is a fine-grained, sandy loam containing approximately 10% clay, 10% silt, and 80% fine sand. The soil has a low water-holding capacity, an organic matter content of <5%, a pH of 8.5 to 11.0, and salinity of 8 to 16 mmhos/cm (*15*). Swelter Shelter is on the main automobile tour through Dinosaur National Monument and is accessed by a short trail.

Archeological excavations conducted at Swelter Shelter in 1964 and 1965 were part of a larger archeological survey of the monument that included many Native American sites (*14*). The inside wall of Swelter Shelter contains Native American petroglyphs and pictographs dating from the Fremont Culture before 1200 A.D. The 1964 and 1965 excavations identified artifacts as old as 7000 to 6000 B.C., as well as two ancient fireplace hearths, one of which contained burned animal bones (*14*). Unknown to those working in 2001, an outbreak of respiratory illness had occurred among those conducting the earlier archeological excavations (*16*).

On June 18, 2001, under the direction of National Park Service archeologists, a team of six student volunteers and two volunteer leaders began work at Swelter Shelter. Work included laying stone steps, building a retaining wall, and sifting dirt for artifacts—an activity that created considerable dust. Within the week before work began, the volunteers and leaders had arrived from their residences throughout the United States; one arrived from Europe. While at the monument, they camped in tents approximately 3 miles away from Swelter Shelter. During June 29 to July 3, all six volunteers, both leaders, and two National Park Service archeologists who worked at the site sought medical care at a local hospital emergency room for acute respiratory and systemic symptoms.

## Methods

### Case Definitions

Persons working at Dinosaur National Monument were defined as meeting the clinical case definition for coccidioidomycosis if they had onset after June 18, 2001, of at least two of the following symptoms: self-reported fever, difficulty breathing, and cough. Persons meeting the clinical case definition were considered to have had laboratory-confirmed coccidioidomycosis if a complement fixation (CF) antibody titer of >1:2 was present or if either of the immunodiffusion tests showed a band of identity. Further confirmation of infection was obtained if there was seroconversion or a >4-fold rise in antibody titer between paired serum samples.

### Cohort Study

From July 2 to 4, 2001, 18 people (all six student volunteers, both volunteer leaders, and all 10 National Park Service archaeologists at Dinosaur National Monument) were interviewed by using a standardized questionnaire to determine symptoms and activities from June 18 to 29. In addition, clinical information was gathered from emergency room and hospital records of persons who sought medical care and recorded on another standardized form. Differences in categorical variables were assessed with the Fisher exact test.

### Laboratory Studies

Acute-phase serum samples were obtained on July 1 or July 3 from persons meeting the clinical case definition and were tested for antibodies to *Francisella tularensis*, *Yersinia pestis*, *Mycoplasma* species, *Histoplasma capsulatum,* and *C. immitis* by using standard techniques at laboratories at the Centers for Disease Control and Prevention. In addition, all persons had serologic tests for *Rickettsia rickettsii,* five for *Legionella*, and five for hantavirus at local laboratories. Blood cultures for bacterial pathogens were obtained during hospitalization.

Convalescent-phase serum samples were obtained from July 16 to 21, 2001. Acute- and convalescent-phase serum samples were assayed for antibodies to *C. immitis* by CF and immunodiffusion (IDCF), primarily to detect immunoglobulin (Ig) G antibodies. Acute-phase serum samples were assayed by immunodiffusion (IDTP) primarily to detect IgM antibodies (IDTP) (*17*); the IDTP assay was further performed with serum concentrated three- to fivefold.

### Environmental Investigation

Monthly average temperature and precipitation data for the Dinosaur National Monument quarry area (approximately 0.5 miles from Swelter Shelter) for 1958 to 2002 were obtained from the Western Regional Climate Center (available from: www.wrcc.dri.edu/cgi-bin/cliMONtavt.pl?utdino and www.wrcc.dri.edu/cgi-bin/cliMONtpre.pl?utdino). Daily rainfall data were obtained from weather station records at the monument.

### Serologic Survey of Monument Workers

From August 15 to 17, 2001, we conducted a serologic survey among Dinosaur National Monument employees to determine the presence of antibodies to *C. immitis.* Because coccidioidomycosis skin test reagents are currently unavailable, testing for antibodies to coccidioidomycosis was performed to assess prior immunity to *C. immitis* among persons who reside or work in the area. Samples were tested using CF and IDCF as described earlier.

## Results

### Cohort Study

**Figure 2:**
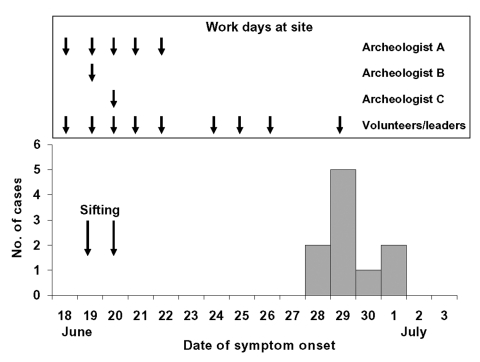
Number of persons meeting the clinical case definition, by date of symptom onset. Days worked at the site are indicated.

Ten of the 18 persons interviewed met the clinical case definition for coccidiodomycosis. The case-patients included all 6 volunteers, both group leaders, and 2 of 10 archeologists. The median age of patients was 17 years; five were male; and all were Caucasian. Illness onsets ranged from June 28 to July 1 (Figure 2).

Because the two group leaders and six volunteers traveled as a group and all became ill, the sites of possible exposure to coccidioidomycosis were limited to Swelter Shelter and their camping area, the only two places visited before June 26. All eight of these persons reported engaging in the same activities at each site; thus, determining specific risk factors at Swelter Shelter or the camping area was not possible. However, among the 10 archeologists, 2 of 3 who worked at Swelter Shelter in June met the clinical case definition compared to none of 7 who did not work there (p = 0.07). The two ill archeologists worked on June 19, when dirt near the petroglyphs was sifted with screens (archeologists A and B, Figure 2). The archeologist who remained healthy (archeologist C, Figure 2) only worked on June 20. On that day, sifting occurred along the trail approximately 15 feet from the petroglyph panel. Sifting did not occur on other days. Therefore, all persons meeting the clinical case definition, and none of the noncase- patients were present at the sifting on June 19 (Figure 2) (p = 0.00002). No archeologist had visited the camping area.

With June 19 being the most likely time of exposure, the median incubation period for the 10 persons who met the clinical case definition was 10 days (range 9–12). These persons reported difficulty breathing (10 persons), nonproductive cough (9 persons), fever (9 persons), fatigue (8 persons), shortness of breath (7 persons), myalgia (6 persons), skin rash (6 persons), and nausea/vomiting (4 persons). Eight persons were hospitalized; the one person who did not report fever had a temperature of 37.8°C on hospital admission. The mean temperature on admission was 38.3°C (range 36.9°C–39.4°C) and the average respiratory rate was 23 per minute (range 18–32). Results of a pulmonary examination were relatively unremarkable except for dry cough. At the time of evaluation, five patients had a maculopapular rash on the neck, trunk, and extremities. The mean leukocyte count at admission was 11,800 mm^3^ (range 5,600–17,700), with an average of 80% neutrophils (range 67%–92%). Results of tests of liver and renal function, including urinalysis, were within normal limits. The average oxygen saturation was 93% (range 88%–97%) by pulse oximetry. Chest radiographs of all 10 case-patients showed bilateral patchy infiltrates. All persons hospitalized were treated with fluconazole and discharged within 3 days.

Acute-phase serum specimens from 9 of 10 persons who met the clinical case definition contained IgM antibodies to *C. immitis,* as determined by IDTP by using concentrated serum samples; one was positive by IDTP before serum concentration. The patient without demonstrable IgM antibodies on convalescent-phase serologic testing had pulmonary infiltrates and a skin rash typical of the other patients. Two of the eight patients with convalescent-phase samples had at least a fourfold increase in CF titer. Initial serologic tests for antibodies to *F. tularensis*, *Y. pestis*, *Mycoplasma* species, *R. rickettsii*, *Legionella,* and hantavirus were negative. Blood cultures were negative for bacterial pathogens.

### Additional Case Investigation

On August 24, state and local health departments jointly recommended that employees minimize soil disturbance and dust inhalation (e.g., by watering down the soil and wearing National Institute for Occupational Safety and Health [NIOSH]–approved N95 respirators) at Swelter Shelter to reduce their risk for *C. immitis* infection. Five persons completed work on the retaining wall and steps on September 24 and 27, 2001. Although the soil was watered periodically during both days, the workers only wore masks on September 24. On October 5, 2001, left shoulder and chest pain, shortness of breath, fever, headache, diaphoresis, and shaking chills without skin rash developed in one worker. A chest radiograph on October 16 indicated left lung pneumonia. Serum samples drawn on October 15 and November 7 demonstrated elevated and rising *C. immitis* IgG and IgM antibody levels by enzyme-linked immunosorbent assays at a commercial diagnostic laboratory, a finding consistent with acute coccidioidomycosis.

### Environmental Investigation

Weather data indicated an unusually long dry period before the 2001 outbreak. No rain fell from May 3 to June 25. For May and June 1958 to 2002, the average temperatures were 14.9°C (standard deviation 1.4) and 20.1°C (standard deviation 1.6), respectively. The average temperatures in May and June 2001 were 17.1°C and 22.5°C. Since 1958, only 1 year (1994) had a warmer average temperature in both May and June.

### Serologic Survey of Monument Workers

Forty employees were enrolled. Their mean age was 43 years; 26 (65%) were male. They had worked at DNM for a median duration of 26 months (interquartile range 9–126 months) and had spent an average of 13.8 years living within 100 miles of the monument. Employee activities included digging up soil or rock (27.5%), sifting sand or dirt (7.5%), and preparing paths or trails (12.5%). A quarter (27.5%) reported work duties that exposed them to dust every day. Serologic tests for *C. immitis* antibodies were negative for all 40 persons.

### Investigation of the 1964–1965 Archeological Team

To investigate the possibility that a similar outbreak occurred during the 1964–1965 archeological excavations at the monument, we interviewed by telephone the leaders of that 25-person, archeological team in January 2003. They identified five team members in whom acute respiratory illness occurred in 1964 to 1965. These five were contacted by telephone; all reported fever, dry cough, and chest pain with onsets toward the end of, or shortly after, the archeological activities. One was hospitalized for 10 days with pulmonary infiltrates; a skin rash and severe arthralgias also developed in this person. Three persons reported subsequent positive skin or serologic tests for *C. immitis*; none had previously lived in an area endemic for coccidiodomycosis. All five ill persons worked at Swelter Shelter in August 1964 or June and July 1965. None of those working at other sites had known illness.

## Conclusion

This outbreak provides evidence of an endemic focus of coccidioidomycosis within Dinosaur National Monument in northeastern Utah, expanding the known geographic distribution of coccidioidomycosis. Multiple factors likely contributed to this outbreak: most patients lived in areas where coccioidomycosis was not endemic and probably were without preexisting immunity to *C. immitis*; the alkalinity of the soil was favorable to the growth of *C. immitis*; a prolonged dry period occurred before the outbreak; and the archeological activities, particularly the sifting of the very fine-grained soil, caused considerable dust exposure.

Several lines of evidence indicate that the endemic focus at Swelter Shelter is highly localized and does not represent a larger unrecognized region of activity in northeastern Utah. First, the serologic survey failed to find recent serologic evidence of *C. immitis* exposure among other Dinosaur National Monument workers, although many reported frequent occupational exposure to soil or dust. However, the serologic survey could not rule out prior exposures since serologic tests are insensitive for past infection (*18*). Second, locally acquired coccidioidomycosis has not been reported among other monument workers or in local residents. Third, although the cause of the 1964–1965 outbreak cannot be definitively determined, the illnesses were remarkably similar to those in 2001 and were only recognized in persons working at Swelter Shelter, despite extensive archeological activities at many other sites (*14*).

Consistent with the hypothesis of a highly focal endemic area at Swelter Shelter, a study of outdoor workers in California indicated that even in disease-endemic areas, highly focal areas of high risk for coccidioidomycosis exist and persist (*19*). Another study showed that after burial of animals that died of coccidioidomycosis in soil free of the organism, repeated isolations of *C. immitis* from the soil at the burial site were possible for at least 7 years, even though soil immediately surrounding the burial site remained uncontaminated (*20*).

The endemic focus at Swelter Shelter may have been established centuries ago. This outbreak and the three that occurred at Native American archeological sites in California outside the known range for *C. immitis* (*3*–*5*) suggest that these sites may represent a unique type of extremely focal endemic area. *C. immitis* is highly concentrated in Native American middens, indicating that the organism was highly prevalent among these people (*21*), and repeated isolations of *C. immitis* have been made at old Native American campsites in endemic areas (*22*). Analysis of the microsatellite loci of *C. immitis* to study the genetic diversity of the fungus has shown that isolates from North America are geographically partitioned. This finding suggests that *C. immitis* was spread throughout the Americas by migrating populations of Native Americans (*23*); the occurrence of outbreaks at Native American archeological sites outside known disease-endemic areas supports this hypothesis. The last identified Native American presence at Swelter Shelter dated before 1200 A.D (*14*).

Why *C. immitis* can apparently persist at Swelter Shelter, an area well outside the known disease-endemic region, is not known. One possibility is that average soil temperatures within the shelter are higher than surrounding areas. Swelter Shelter is the only known Native American site at Dinosaur National Monument that faces south, is relatively sheltered, and receives considerable reflected heat from surrounding rock.

Attack rates during outbreaks at Native American sites outside of disease-endemic areas have been high. During the three California outbreaks, attack rates ranged from 48% to 77% (*3*–*5*), compared to 91% among those who worked at Swelter Shelter. At the shelter, sifting of dirt with screens appeared to place persons at a particularly high risk; the 91% attack rate in this outbreak probably reflects the fact that all of the workers participated in or close to the sifting.

We conclude that a newly identified endemic focus for *C. immitis* exists at a Native American archeological site in northeastern Utah. Archeologists working at Native American sites in the western United States are at risk for coccidioidomycosis, even outside recognized disease-endemic regions. Sifting of dirt during archeological activities may put persons at a particularly high risk for exposure to *C. immitis*. Measures to decrease dust and aerosolization of spores, such as soil wetting, and the use of appropriate masks while performing archeological work at these sites may reduce the risk. Archeological workers might benefit from vaccination; efforts are currently focused on developing a vaccine for coccidioidomycosis (*24*)
